# Optimizing the operational conditions for microalgae biomass drying using tray dryers

**DOI:** 10.1038/s41598-025-34616-w

**Published:** 2026-01-29

**Authors:** R. López Pastor, M. G. Pinna-Hernández, J. A. Sánchez Molina, J. L. Casas López, M. I. Maldonado Rubio, F. G. Acién Fernández

**Affiliations:** 1 Solar Energy Research Centre (CIESOL), Joint Centre University of Almería-CIEMAT, Almería, 04120 Spain; 2 Department of Chemical Engineering, University of Almería, Carretera de Sacramento s/n, La Cañada de San Urbano, Almería, 04120 Spain; 3 Department of Informatics, University of Almería, Carretera de Sacramento s/n, La Cañada de San Urbano, Almería, 04120 Spain; 4 CIEMAT-Plataforma Solar de Almería, Ctra. De Senés s/n, Tabernas, Almería, 04200 Spain

**Keywords:** Modelling, Biomass degradation, Drying kinetics, Pre-treatment, Energy science and technology, Engineering, Environmental sciences

## Abstract

Drying is a critical yet energy-intensive step in the valorization of microalgae biomass, essential for ensuring long-term stability and enabling downstream processing. This study investigates the technical feasibility and performance of tray drying as an alternative to conventional methods, using *Chlorella* sp. biomass. Experiments were conducted at drying temperatures ranging from 60 to 80 °C and biomass layer thicknesses between 0.3 and 1.0 cm, simulating industrial tray dryer conditions. Drying kinetics were assessed through moisture ratio and water content conditions, with a power-law model applied to describe the drying rate as a function of moisture content. The results demonstrated that thinner layers and higher temperatures significantly reduced drying time, with full dehydration within 5 h at 80 °C and 0.3 cm thickness. However, spectrophotometric analysis revealed a trade-off between drying efficiency and biomass quality, with pigment degradation increasing with temperature and time. A polynomial model was developed to predict pigment deterioration based on operational parameters. These findings provide a robust foundation for the design and scale-up of tray drying systems and offer a practical framework for optimizing the balance between process efficiency and product quality in microalgae biorefineries.

## Introduction

Microalgae biomass holds great potential for high-value applications in pharmaceuticals, cosmetics, food, feed, agriculture, and bioenergy^1–3^. While all stages of the production chain—from culture medium preparation to bioreactor operation—impact the overall process efficiency and cost, harvesting and downstream processing remain critical bottlenecks. These steps alone can account for over 30% of the total production cost^4^, yet they have historically received less attention compared to cultivation technologies. Although recent advancement have focused on nutrient reuse and reactor design^5,6^, optimizing harvesting and processing technologies is now essential to enhance economic viability and unlock large-scale deployment^7–9^.

Harvesting and dewatering are indispensable steps in microalgae processing, as they enable the transition from dilute cultures (typically < 1 g/L) to concentrated biomass suitable for downstream applications^7,10^. However, the resulting concentrated biomass remains unstable due to its high-water content and susceptibility to microbial and enzymatic degradation. Therefore, stabilization methods are essential to preserve the functional, nutritional, and physicochemical properties of the biomass during storage, transport, and further processing. Among available techniques, drying is the most widely applied, as it effectively reduces water activity and extends shelf life. Nevertheless, it also represents one of the most energy-intensive and cost-demanding steps in the microalgae value chain^8,11^, especially when using freeze or spray drying, which can also compromise thermolabile compounds such as pigments and antioxidants^12^. Despite its pivotal role in biomass valorization, stabilization through drying has traditionally received less attention than cultivation strategies. Yet, improving drying efficiency while preserving biomass quality is now a key challenge for economically viable and scalable microalgae-based bioproducts^13,14^. Developing cost-effective and energy-efficient drying solutions that minimize quality losses is crucial to support the sustainable growth of the algae biotechnology sector.

Recent reviews have reconfirmed that drying remains one of the most energy-intensive and quality-limiting steps in microalgae processing, highlighting the advantages and constraints of conventional, convective, freeze, spray, microwave and hybrid drying technologies^15^. A previous studies have shown that harvesting and drying are among the most energy- and cost-intensive steps in microalgae processing, with operational costs ranging from 0.5 to 2 €·kg⁻¹ and energy consumption from 0.2 to 5 kWh·kg⁻¹ for open cultivation systems. These figures are somewhat lower for closed photobioreactor systems, where costs range from 0.1 to 0.6 €·kg⁻¹ and 0.1 to 0.7 kWh·kg⁻¹, respectively^8^. Among the available drying technologies, freeze dryers and spray dryers are the most employed at industrial scale due to their ability to produce stable, high-quality biomass. However, both systems involve significant capital and energy requirements. Freeze drying, while preserving bioactive compounds, demands substantial investment and high energy input for vacuum and low-temperature operation^16^. Spray drying, while faster and more scalable, involves but consumes large amounts of energy and can lead to thermal degradation of sensitive compounds, thus compromising biomass quality^17^. Updated assessments of spray-drying performance confirm that, despite its scalability and rapid moisture removal, this technique can induce significant thermal degradation of pigments and other sensitive biomolecules in microalgae^18^.

Solar dryers present another alternative, particularly in regions with abundant solar irradiance. Passive solar drying significantly reduces the cost of biomass drying. In trials conducted inside a greenhouse at ambient temperature, it was observed that the moisture content of tomato and pepper plant residues could be reduced to below 30% within 30 days, and in the case of cucumber in June, within 17 days. This study also showed that the biomass layer height in the drying tray was a key parameter influencing the drying rate, with thinner layers requiring less drying time. Nevertheless, the long drying periods associated with passive solar drying would not be feasible for microalgae^19^. Indirect solar dryers, which use solar-heated air rather than direct sunlight, offer a more controlled environment compared to open drying. These systems can incorporate fan heaters, dehumidifiers, and ventilation units to support the drying process^20–22^. However, challenges remain, particularly in maintaining consistent temperature and humidity conditions, which are critical for preserving sensitive compounds. Recent advances in hybrid solar–air systems have shown improvements in thermal efficiency, yet maintaining stable drying conditions remains challenging and strongly limits their applicability for high-value microalgal biomass^21^. As a result, the use of solar dryers for high-value applications is still limited. Although recent studies have demonstrated the feasibility of preserving biomass quality under indirect solar drying conditions^23^, the overall energy efficiency and process controllability still require significant improvements. Optimizing these low-cost alternatives is essential to enable the sustainable and scalable production of microalgae-based bioproducts across a wider range of applications.

In this context, tray dryers (oven-type) are commonly employed in small- to medium-scale microalgae production due to their affordability, ease of use, and widespread availability in laboratory and pilot facilities. These systems offer a practical solution for biomass stabilization where advanced equipment like freeze or spray dryers is not economically viable. However, their application to microalgae drying remains largely empirical, with operational parameters such as drying temperature, biomass layer thickness, and airflow often selected arbitrarily or based on general food-drying practices. This lack of process standardization leads to suboptimal energy use, extended drying times, and potential degradation of valuable biomass components^24,25^. Crucially, there is a notable absence of robust experimental data and validated kinetic models tailored to microalgae, which hinders the rational design and scale-up of tray drying processes. Recent kinetic studies on *Chlorella vulgaris* have demonstrated the importance of using species-specific drying models to accurately describe moisture diffusion behavior and guide scale-up strategies^26^. Unlike other drying technologies, tray dryers have not been systematically studied for their performance with microalgal biomass, particularly in terms of heat and mass transfer behavior, drying kinetics, and product quality retention. This knowledge gap limits their potential use in commercial operations and highlights the need for research focused on developing predictive models and simulation tools that enable efficient process design, scale-up, and integration into microalgae biorefineries^27^.

The objective of this work is to analyze and model the drying process of microalgal biomass obtained demonstration plan cultivation microalgae (1000 m^2^) in tray dryers, proposing this technology as a suitable alternative for small- and medium-scale production facilities. The study aims to provide a comprehensive understanding of the physical phenomena involved during microalgae drying (material no study with technology proposed) —particularly heat and mass transfer mechanisms—as well as to identify the operational conditions that enable efficient moisture removal while preserving the biomass quality. Through a combination of experimental trials and mathematical modeling, the research seeks to define optimal parameters such as drying temperature and layer thickness, and to develop predictive tools that support process optimization. Ultimately, this work contributes to the development of drying strategies that not only enhance process efficiency but also minimize the degradation of heat and oxidation sensitive compounds, thereby ensuring the retention of the biomass functional and nutritional properties.

## Materials and methods

### Biomass production and harvesting

The microalgal biomass used in this study was obtained from the *Chlorella* sp. strain cultivated in a 1,000 m² raceway pond located at the SABANA demonstration facility (CIESOL-IFAPA Research Center, La Cañada de San Urbano, Almería, Spain). The culture was operated in continuous mode at a dilution rate of 0.2 day⁻¹, using freshwater and commercial fertilizers as the nutrient source for medium preparation. The pH was tightly regulated at 8.0 through on-demand injection of pure CO₂, ensuring optimal growth conditions and promoting carbon uptake. To prevent oxidative stress, dissolved oxygen levels were maintained below 200% Sat. via intermittent aeration, activated when thresholds were exceeded. The culture temperature was not controlled but followed ambient environmental conditions, fluctuating between 18 °C and 24 °C throughout the biomass production period. Once harvested, the biomass was concentrated by centrifugation using a GEA Westfalia Separator OSC-20 continuous-flow centrifuge, which allowed for efficient dewatering. The resulting concentrated biomass was used for drying tests.

### Drying tests

Drying tests were developed with a model ED 23 natural convection tray dryer (BINDER-GmbH, Germany). The tray dryer operated under natural convection, with air velocities approximately 0.2 m·s⁻¹ during all experiments, passive venting, and no independent humidity control; moisture removal occurred as humid air was gradually released through the fixed exhaust port. The tray dryer characteristics allow the use of identical drying conditions inside regardless of the size or quantity of sample. In addition, its thermal insulation reduces operating costs and energy losses. The objective of the tests was to reproduce conditions similar to those encountered in industrial tray drying systems, particularly in terms of temperature and sample thickness. Microalgae biomass used in the tests was previously concentrated by centrifugation, resulting in samples with a total solids content below 20%, corresponding to moisture levels above 80%. Drying trials were conducted at controlled temperatures ranging from 60 °C to 80 °C, while varying the thickness of the biomass layer between 0.3 and 1.0 cm. Samples were placed in individual aluminum trays (10 cm in diameter), with up to three replicates per experimental condition to ensure reproducibility. During each experiment, trays were removed from the oven and weighed at hourly intervals to monitor the progress of water removal. These periodic measurements allowed for the determination of both moisture loss and drying rate over time.

The drying velocity was calculated using Eq. 1, where W is the drying rate in kg of water removed per square meter per hour, S is the mass of dry solids in kg, A is the surface area of the tray in m², and -dX/dt is the time derivative of the water content (kg of water per kg of dry biomass).

1 $$\:W=(S/A)\times\:(-dX/dt)$$

Energy consumption was quantified using an external digital power meter, and the specific value of 0.66 kWh kg⁻¹ of evaporated water was obtained by dividing the recorded electrical energy input by the experimentally measured mass of water removed during drying.

### Assessment of biomass quality

To evaluate the impact of drying conditions on the quality of the microalgal biomass, samples were taken from one of the replicates at specific time intervals during each experiment. The collected biomass samples were appropriately diluted and analyzed using a UV-Vis spectrophotometer (GENESYS 10 S UV-Vis, Thermo Scientific) to obtain absorbance spectra across a defined wavelength range. This non-destructive analytical technique enables the detection of changes in the optical properties of the biomass, which are indicative of chemical and structural alterations occurring during the drying process.

In microalgae, the absorbance spectrum is closely associated with key compounds such as pigments (e.g., chlorophylls, carotenoids) and other biomolecules sensitive to heat and oxidation. Therefore, significant deviations in the spectral profile compared to the fresh (undried) sample indicate a loss of functional or nutritional quality due to degradation. To quantify these spectral differences and assess the extent of biomass deterioration, the Mean Absolute Error (MAE) was calculated with respect the absorbance spectra of initial samples. The MAE provides a single value representing the average absolute difference in absorbance across the measured wavelength range and is defined by Eq. 2, where Abs represent the absorbance values at each wavelength for the drying and initial samples, respectively. A higher MAE value corresponds to greater spectral deviation and thus greater biomass deterioration. This metric allowed for a systematic comparison of the effects of different drying conditions and facilitated the identification of operational parameters that minimize quality loss during tray drying of microalgae.

2 $$\:MAE=\frac{1}{n}\sum\:{\int\:}_{400}^{700}\sqrt{{\left({Abs}_{drying}-{Abs}_{initial}\right)}^{2}}$$

## Results and discussion

The preservation of microalgal biomass is a critical step in its valorization for diverse applications across the food, pharmaceutical, and bioproduct industries. Effective drying methods are essential for reducing water activity, thereby inhibiting microbial proliferation and enzymatic degradation, which are key to maintaining product stability and extending shelf life. Furthermore, adequate drying reduces biomass volume and weight, facilitating more efficient storage and transportation. Among the various drying technologies, tray drying is frequently employed due to its operational simplicity, relatively low capital investment, and adaptability for processing biomass at small to pilot scales. However, applying tray drying to microalgae presents unique challenges, primarily due to the thermosensitive nature of many valuable microalgal biocompounds. Therefore, optimizing drying parameters is paramount to achieve maximal moisture reduction while safeguarding high-value components such as proteins, pigments (e.g., phycocyanin), and lipids^23,28^.

The initial moisture content of microalgal biomass is the first critical variable influencing the drying process. This parameter exhibits considerable variability, primarily contingent upon the dewatering technique employed. For instance, continuous centrifugation typically yields microalgal biomass with a high moisture content, often reaching up to 90%, whereas batch centrifuges or systems integrating filtration can achieve more substantial reductions to approximately 70%^8^. Biomass with a moisture content below 70% tends to exhibit rheological properties characteristic of a semi-solid or paste, which complicates downstream handling, particularly for efficient pumping or uniform spreading onto drying trays in industrial setups^29,30^. For these reasons, an initial moisture content of 80% is considered optimal, ensuring sufficient flowability and homogeneous distribution on drying surfaces for consistent moisture removal.

Once the initial moisture baseline is established, the next crucial step involves optimizing the operational conditions within the tray drying system. Key parameters governing the drying process include drying temperature, air velocity, and the layer thickness of the biomass on the trays. While air flow is often a fixed parameter in commercial drying ovens, typically ranging between 1 and 2 m/s, the drying temperature and biomass layer thickness remain the primary variables that can be precisely tuned to enhance drying efficiency and product quality. In the present study, drying temperatures of 60 °C, 70 °C, and 80 °C were systematically investigated. This range aligns with optimal values reported in literature, where successful microalgae drying has been achieved between 40 °C and 100 °C, depending on species-specific heat sensitivity and targeted end-use^15,31^. To further reduce drying time and enhance thermal transfer, the thickness of the microalgae “cake” was also varied 0.3, 0.6 and 1.0 cm). Thinner layers are known to facilitate more efficient moisture diffusion and heat penetration, consistent with prior findings on improving drying kinetics in tray-based systems^24^. These controlled variations are essential for understanding their synergistic effects on drying performance and the preservation of microalgal biomass quality.

**Fig. 1:**
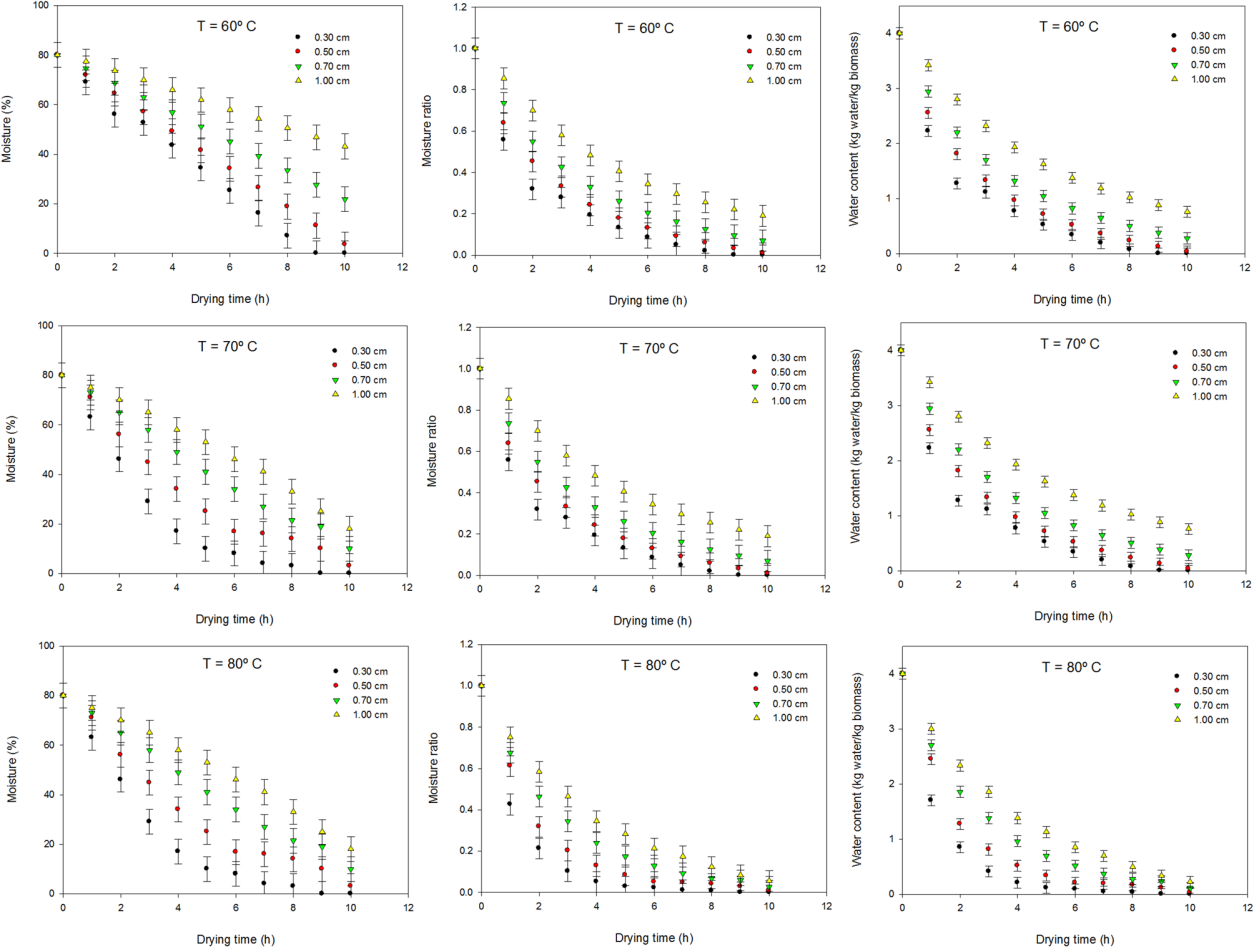
Variation of water content of samples with time as a function of temperature (60, 70 and 80° C) of the air and thickness of the biomass sludge (0.3, 0.5, 0.6 and 1 cm).

The drying kinetics of microalgal biomass, expressed as the progressive decrease in moisture content (% w/w) over time, consistently reflected typical behavior observed in thermally assisted dewatering, driven by coupled heat and mass transfer phenomena^26^. The rate of moisture removal, evidenced by the slope of the drying curves, was significantly influenced by both cake thickness and drying temperature (Fig. 1). Notably, thinner biomass layers and higher temperatures resulted in steeper slopes, indicating more rapid moisture loss and consequently shorter drying durations^26,32^. This acceleration of drying rate with increased temperature and reduced thickness is a well-established principle in convective drying, as higher temperatures enhance the vapor pressure difference and thinner layers reduce internal resistance to moisture diffusion^33^. Under the least efficient condition tested—60 °C and a 1.0 cm thick biomass layer—samples failed to reach the desired < 5% final moisture content even after extended drying periods. Full dehydration at this temperature was only achieved when the cake thickness was 0.5 cm or thinner, requiring up to 10 h. Conversely, under the most favorable drying condition of 80 °C, biomass layers ≤ 0.7 cm reached complete dryness in under 7 h. The most efficient scenario was achieved at 80 °C and 0.3 cm thickness, where drying was completed in just 5 h—a reduction of up to 5 h compared to the slowest scenario. Comparable drying times were documented when processing biomass of *Scenedesmus*, where drying time at 80 °C dropped to 6–8.5 h for thin layers^24^. Higher temperatures increase the vapor pressure gradient between the biomass surface and the drying air, thus accelerating convective mass transfer. In parallel, thinner biomass layers reduce the internal diffusion path for moisture migration inside the cake, lowering internal mass-transfer resistance. Together, these mechanisms enhance heat transfer efficiency and moisture diffusion, resulting in faster drying rates and steeper drying curves. These findings underscore the critical role of optimizing both temperature and layer thickness to achieve efficient and complete dehydration of microalgal biomass, aligning with observations that optimal drying temperatures for microalgae often fall within the 60–80 °C range to balance drying efficiency and quality preservation^34^.

While total moisture content (% w/w) is a standard metric for assessing sludge consistency after harvesting, the drying science literature favors more nuanced and analytically rigorous descriptors for kinetic analysis. Two widely applied metrics are the Moisture Ratio (MR) and the Water Content (X), also known as dry basis moisture content. The Moisture Ratio (MR) is a dimensionless parameter normalized from 1.0 at the onset of drying to 0 at equilibrium, enabling standardized comparison across different materials. Water Content (X) quantifies the mass of water per unit mass of dry solids (e.g., kg_water_/kg_dry solids_), offering a direct interpretation of drying progress and capacity, particularly useful for mass balance calculations. In this study, both MR and X were calculated from empirical drying data and are presented in Fig. 1. The drying curves for both metrics exhibited classic exponential decay trends, characteristic of the falling rate period where moisture diffusion governs the drying process. Initial values were observed at MR = 1.0 and X ≈ 4.0 kg_water_/kg_dry solids_, gradually approaching zero as the biomass reached its equilibrium moisture content. At the start of the drying process, the initial water content was approximately 4 kg_water_/kg_dry solids_, which is consistent with typical values reported for centrifuged or filtered microalgae biomass. Initial water contents in the range of 3.8 to 4.2 kg_water_/kg_dry solids_ have been reported when using rotary drying systems with inert beds^35^. In this study, final water content values approached 0.0 kg_water_/kg_dry solids_, reflecting nearly complete dehydration under optimal conditions. For the moisture ratio (MR), our exponential decay profiles closely resemble those previously modeled using Page or Henderson-Pabis equations. Specifically, initial MR values of 1.0 declining to below 0.1 by the end of the drying cycle were also reported in thin layer drying experiments of microalgae paste^27^ and in oven drying of *Chlorella pyrenoidosa*^25^, indicating a strong agreement in moisture reduction kinetics.

**Fig. 2:**
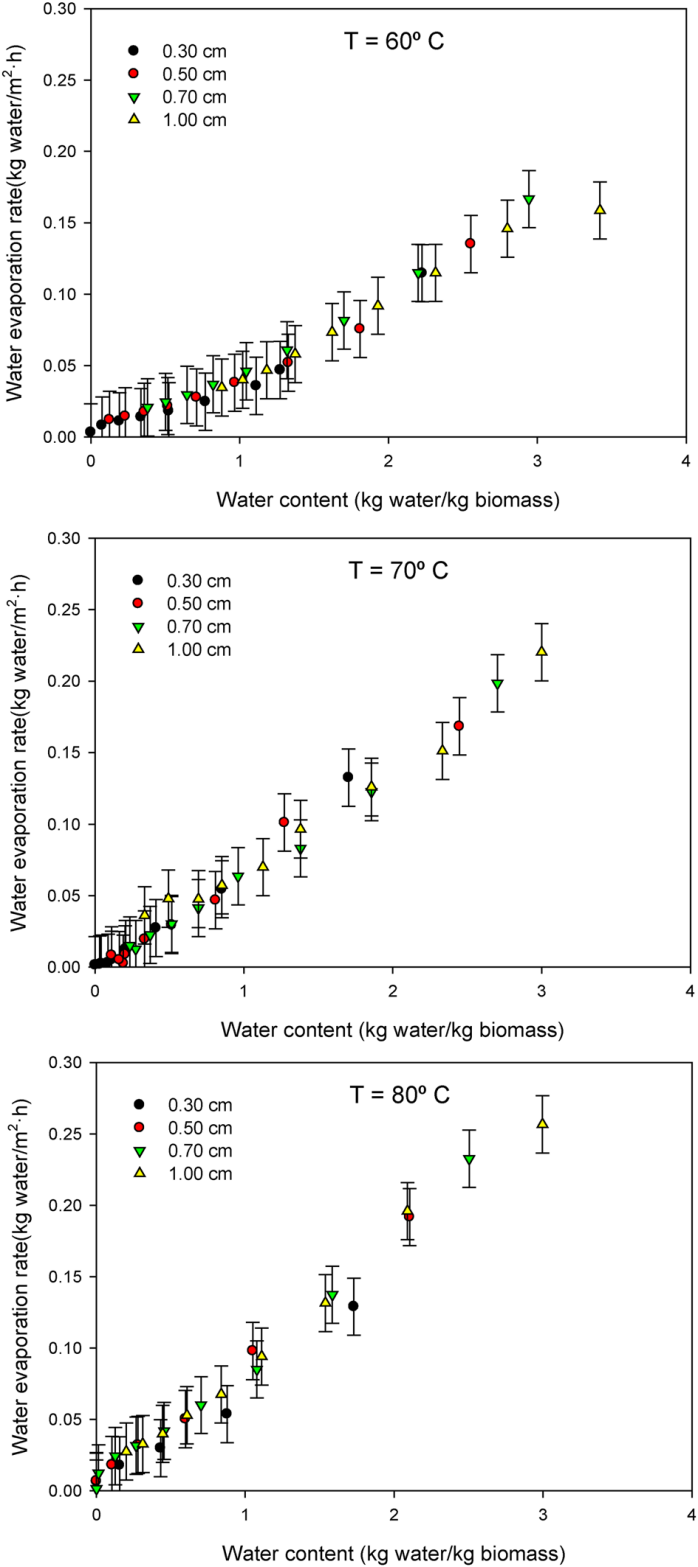
Variation of water evaporation rate per unit surface as a function of water content of the cake (0.30, 0.5, 0.7 and 1.0 cm) at the temperatures essayed (60, 70 and 80 °C).

For analysis and modeling purposes, the water evaporation rate (kg_water_/m^2^·h) is the critical parameter. This metric is fundamental for defining the size of drying equipment for a determined processing capacity, as it directly correlates with the total area equivalent needed for efficient operation. The water evaporation rate was calculated from experimental measurements by monitoring the mass loss from the biomass cake over time, considering the known surface area. This rate is primarily determined by the drying conditions within the oven, including drying air properties (temperature, humidity) and fluid dynamics (air velocity, turbulence), as well as the intrinsic characteristics of the biomass cake that can either facilitate or hinder water evaporation. Since the operational conditions in the oven (except temperature) remained constant during the experiments, variations in the water evaporation rate were primarily studied with respect to the water content of the cake. Results show that the water evaporation rate decreased as the water content of the cake diminished, with the specific variation differing across the three temperatures tested (Fig. 2). Notably, the water evaporation rate was found to be independent of the cake thickness, irrespective of the temperature. Typically, drying curves exhibit two distinct zones: an initial constant rate period, corresponding to the evaporation of free water from the material’s surface, followed by a falling rate period where the drying velocity reduces as internal moisture diffusion becomes the limiting factor. Results indicate the absence of a distinct constant rate period, suggesting that no free water is readily available on the cell surface, and the entire material behaves like a gel. This behavior is common in materials with high initial moisture content where internal resistance to mass transfer quickly becomes dominant^33^. Consequently, the main mechanism limiting the drying process in this study was the diffusion of water within the sample.

**Fig. 3:**
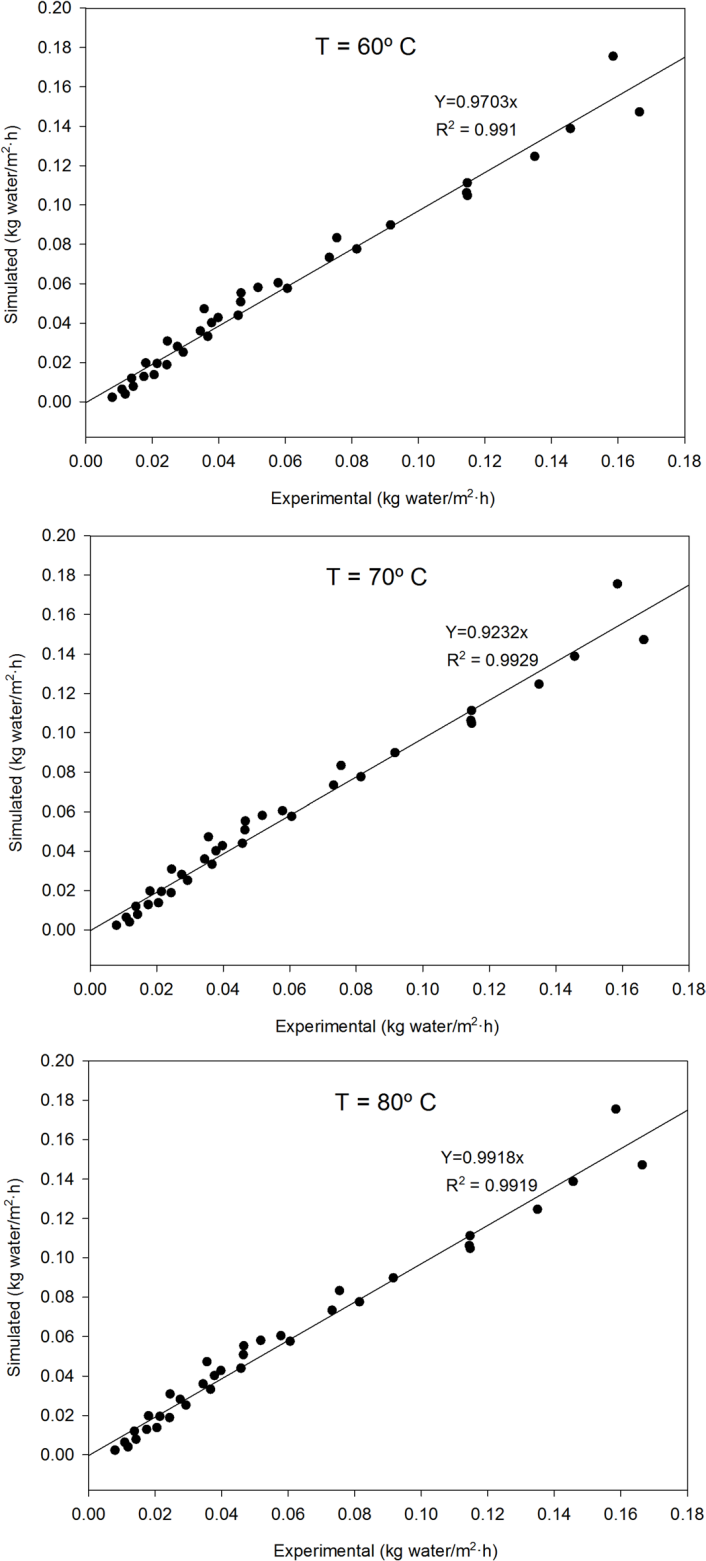
Correlation between experimental values of drying velocity and simulated data using the proposed model. Considered three temperatures (60, 70 and 80 °C).

To further characterize this phenomenon, various mathematical equations have been proposed for modeling drying processes, particularly focusing on the falling rate period where internal mass transfer limits moisture removal, such as the widely used Page model^36^ and other common models like the Newton and Henderson-Pabis models^37,38^. Among these, the potential function is one of the most widely used due to its ability to accurately represent the variation of the water evaporation rate as a function of the water content of the cake. This function is expressed as:

3 $$\:W=a\cdot{X}^{b}$$

where ‘a’ and ‘b’ are characteristic parameters of the model, and ‘X’ is the water content of the cake (kg_water_/kg_dry solids_). Data from the experiments performed at the three temperatures were fitted to this equation to confirm its validity in representing the behavior of the system and to obtain the values of the characteristic parameters of the model. Figure 3 demonstrates that the proposed model fits all the experimental data sets accurately, with the specific values of the fitting parameters presented in Table 1. Different values for the fitting parameters were found across the tested temperatures, which is expected as temperature influences both the intrinsic properties of the cake (e.g., water diffusivity, density) and the properties of the drying air, even when the oven is operated at consistent fluid dynamic conditions.

**Table 1:** Values of characteristic parameters of the model proposed for drying in trays.

Temperature, ºC	60	70	80
a	0.0414	0.0604	0.0861
b	1.1721	1.1000	1.0209

**Fig. 4:**
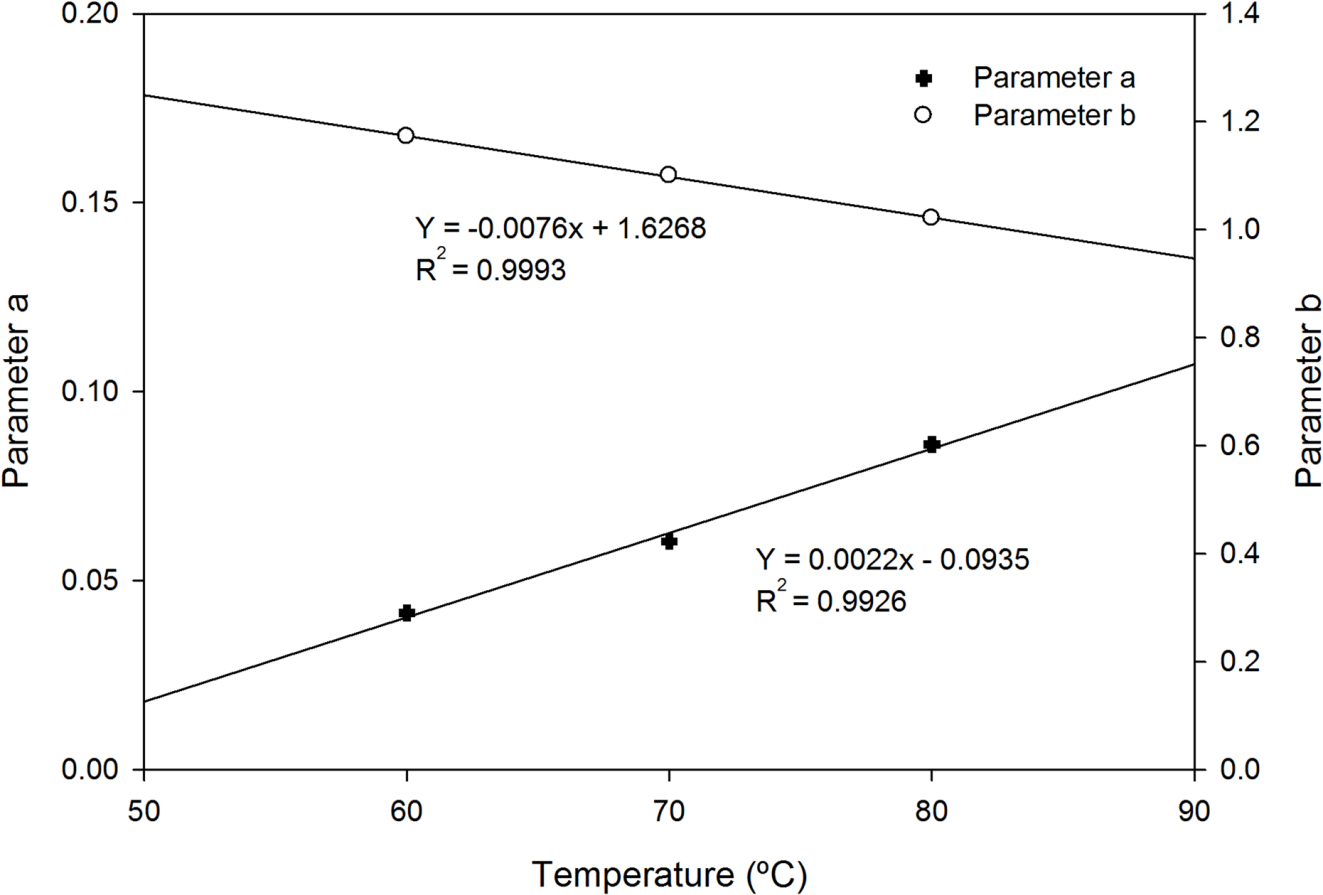
Variation of fitting parameters a and b with temperature (60, 70 and 80 °C) in the oven during the drying process. Linea correlation allows to estimate these parameters for simulation purposes in the range of operational conditions tested.

This power-law relationship for drying rate has been successfully applied to various biological materials, including fruits and vegetables^39,40^, demonstrating its versatility in describing the falling rate period where moisture movement is diffusion-controlled. The analysis of the influence of temperature on the fitting parameters reveals a linear correlation for both ‘a’ and ‘b’ (Fig. 4). Specifically, parameter ‘a’ increases with temperature, whereas parameter ‘b’ decreases with temperature. The slope of variation for parameter ‘b’ with temperature was observed to be higher than that for parameter ‘a’. This indicates that temperature variations have a larger effect on the exponent (‘b’) of the exponential equation utilized, signifying a more pronounced impact on the rate at which the drying velocity diminishes with decreasing water content. While more research is needed to better understand and model the underlying phenomena behind these variations, these linear equations are sufficient for modeling and simulation purposes, allowing for the optimization of operational conditions for the drying process.

In addition to the drying kinetics, which determine the required time and equipment size for a given processing capacity, the energy consumption represents a key parameter influencing both the efficiency and cost of microalgae drying. Theoretically, the latent heat of vaporization establishes a minimum energy requirement of approximately 0.63 kWh kg⁻¹ of water evaporated. Experimental measurements obtained in this study yielded an average specific energy consumption of 0.66 kWh kg⁻¹, a value remarkably close to the theoretical limit. This close agreement demonstrates the high thermal efficiency of the tray dryer, where most of the supplied heat was effectively used for water evaporation rather than lost to the environment. As the oven maintained a constant internal temperature through controlled heat supply, the evaporation rate at the biomass surface was identified as the main factor determining total energy demand. The system’s robust thermal insulation minimized heat losses, confirming that most of the input energy was converted into latent heat. Similar energy consumption ranges (0.6–1.1 kWh kg⁻¹ H₂O) have been reported for convective and drum drying systems, which are considered among the most efficient conventional drying techniques for microalgae when compared to spray drying (≈ 1.09 kWh kg⁻¹ H₂O). Furthermore, comparative techno-economic analyses of solar-assisted drying systems indicate that optimized air–solar configurations can achieve specific energy demands as low as 1.64 kWh kg⁻¹ H₂O, reducing operational costs by over 50% compared to fossil-based drying^22^. These values reinforce that the energy performance obtained in the present study is close to the best-case scenario reported in literature, validating the effectiveness of tray drying for small- to medium-scale biomass stabilization. Considering an average electricity price of €0.155 kWh⁻¹, the corresponding drying cost can be estimated at €0.10 kg⁻¹ of water evaporated. Given an initial moisture content of about 4 kg H₂O kg⁻¹ dry biomass, the final drying cost amounts to roughly €0.41 kg⁻¹ of biomass processed. This cost is consistent with values reported for efficient convective systems and highlights that, when properly optimized, tray drying can provide a cost-effective and energetically rational solution for microalgae biomass preservation^22,41^.

**Fig. 5:**
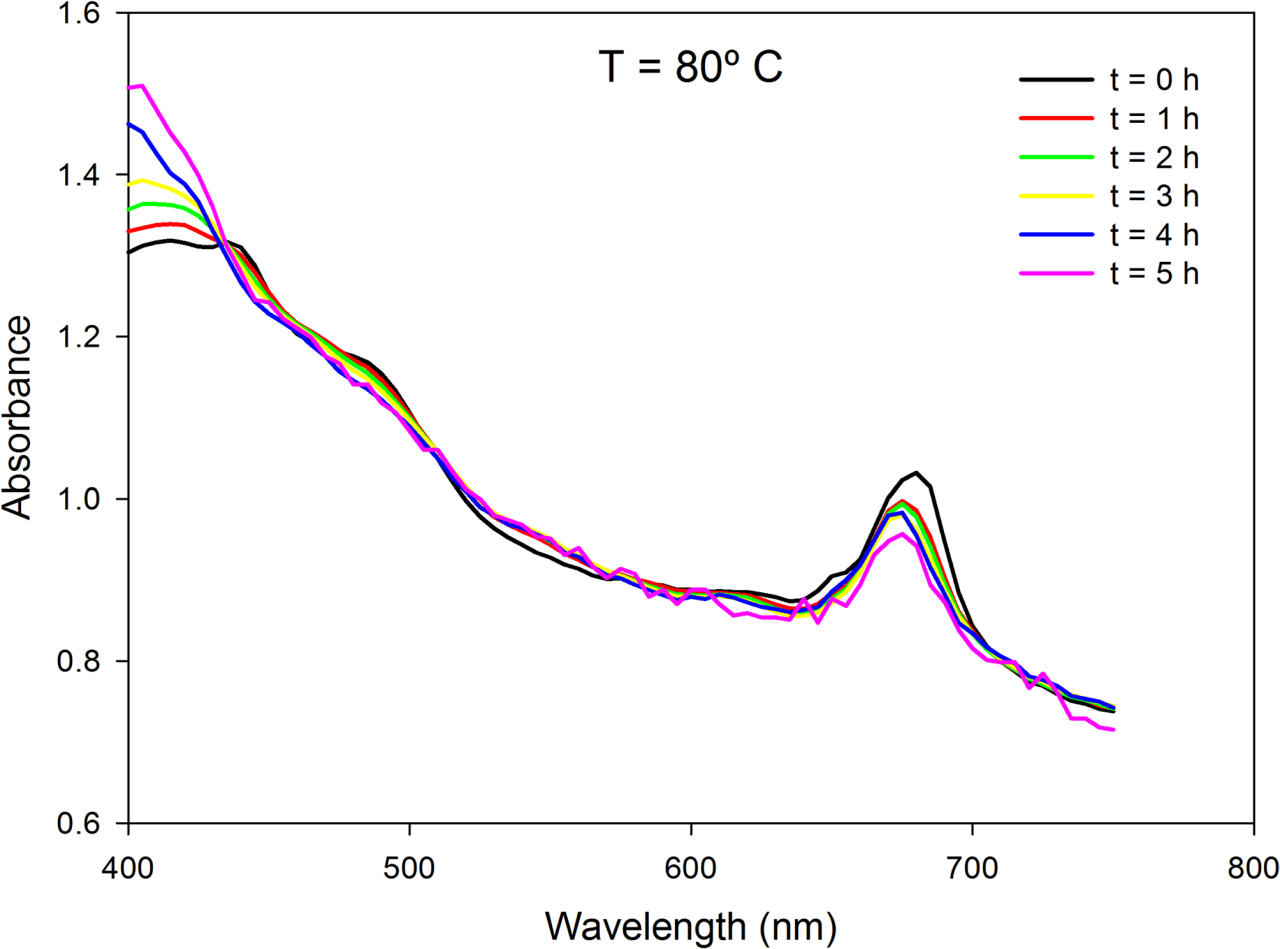
Absorbance spectrum of the biomass at different times (0–5 h) during the drying process at 80 °C.

Beyond drying kinetics, assessing the deterioration of biomass quality during the drying process is crucial for industrial applications. Different methods are employed to evaluate this deterioration, including direct chemical determination of specific compounds (e.g., proteins, lipids, carbohydrates, pigments), evaluation of changes in antioxidant capacity, and analysis of variations in spectrophotometric properties^42,43^. In this study, a rapid method for evaluating the changes in the absorption spectra of the samples was employed to quantify biomass deterioration. Figure 5 illustrates the variation of the absorption spectrum when drying a 0.5 cm thick cake at 80 °C for up to 5 h. It is concluded that while the overall absorption spectrum does not largely modify under these conditions during the process, certain discernible changes do occur, particularly at wavelengths corresponding to key pigments such as chlorophylls (680 nm) and carotenoids (450 nm). These spectral shifts are indicative of pigment degradation, a common issue during thermal processing of microalgae, often linked to the loss of nutritional and functional quality^42,44^.

**Fig. 6:**
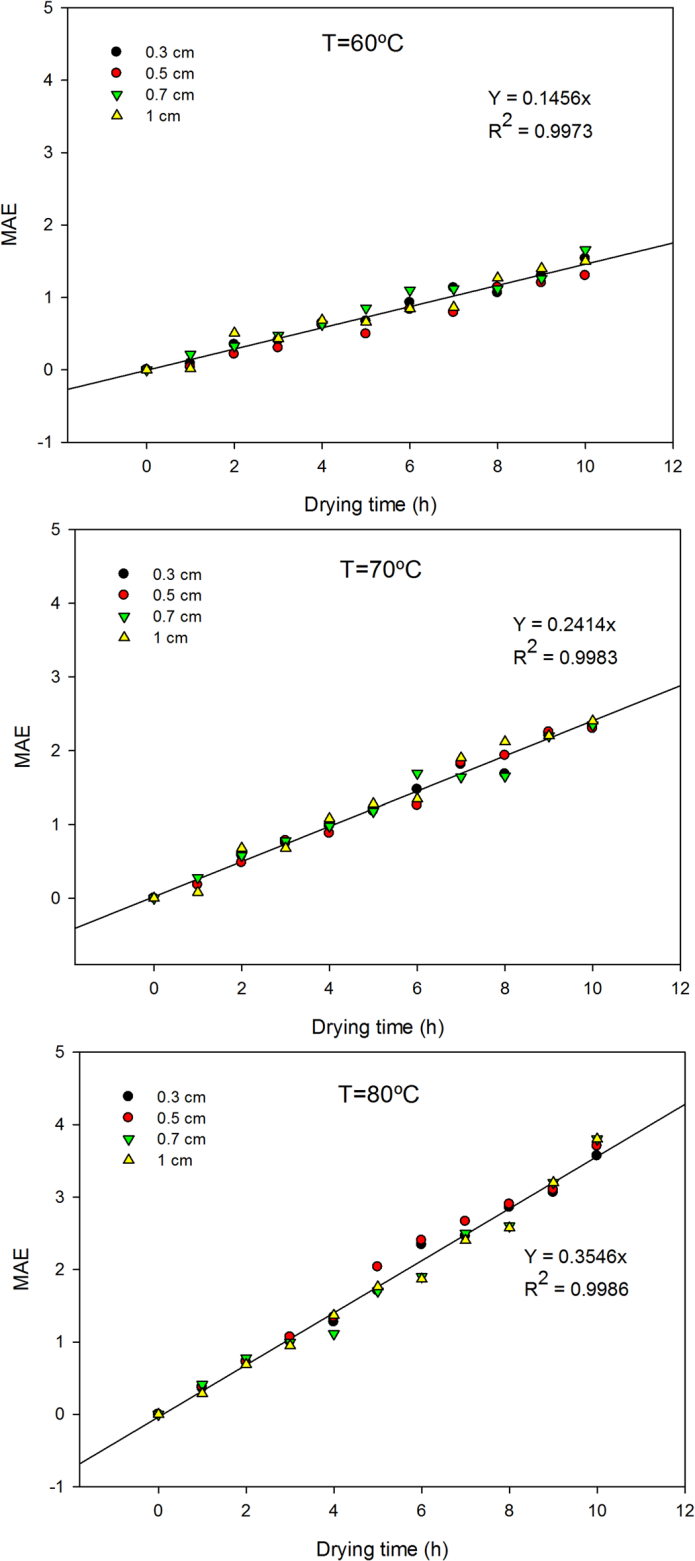
Variation means absolute error of absorbance spectra with the time of drying as a function of drying conditions: temperature: 60, 70 and 80 °C and cake thickness: 0.3, 0.5, 0.7 and 1 cm.

To quantitatively assess the extent of this spectral variation, the Mean Absolute Error (MAE) was calculated with respect to the absorption spectrum of the original, undried sample. Results show that the mean absolute error increases with higher drying temperatures, indicating greater biomass deterioration (Fig. 6). Furthermore, MAE also increases with drying time, suggesting that prolonged exposure to heat contributes to degradation. However, no significant variations in MAE were observed according to the thickness of the cake, implying that for the range tested, cake thickness primarily affects drying rate rather than the extent of deterioration under these specific conditions. Statistical analysis of the data confirms this behavior. From ANOVA, the P-value was 0.0000 for both temperature and time variables, unequivocally demonstrating their statistically significant impact on biomass deterioration. In contrast, the P-value for thickness was as high as 0.9923, indicating no statistically significant effect. Moreover, temperature exhibited a higher F-ratio value of 117.11 versus a value of 80.73 for time, signifying a more pronounced effect of drying temperature on the deterioration of biomass quality. This observation is consistent with other studies on microalgae, where higher drying temperatures have been shown to cause more significant degradation of heat-sensitive compounds like pigments and proteins. For instance, oven-drying led to a greater reduction in antioxidant potential, pigment, and protein content in macroalgae compared to freeze-drying, emphasizing the detrimental effects of elevated temperatures^12^. Similarly, degradation tendencies for pigments and tocopherols in microalgae after thermal drying treatments has been also reported^14^. To provide a predictive tool for quality assessment, a polynomial function was fitted to correlate the deterioration of the biomass (expressed as MAE) with the drying conditions (Eq. 4):

4 $$MAE\,=\, - \,0.0011 \cdot T\, - \,0.4991 \cdot t\,+\,0.0106 \cdot t \cdot T\, - \,1.45 \times {10^{ - \,5}} \cdot {T^2}\,+\,6.29 \times {10^{ - \,4}} \cdot {t^2}$$

**Fig. 7:**
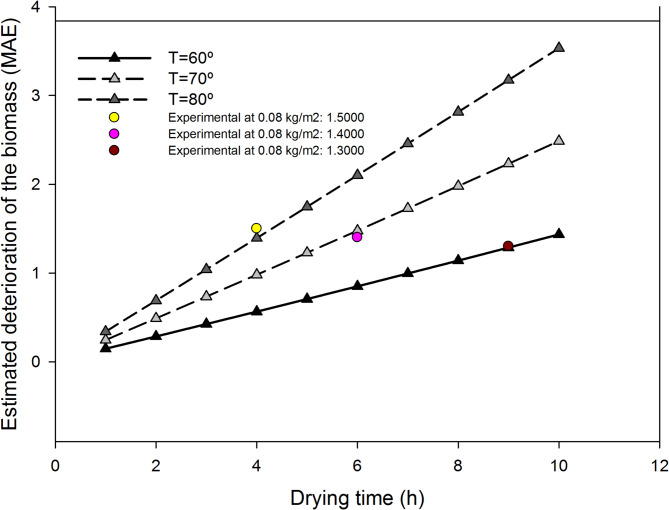
Estimated deterioration of the microalgae biomass as a function of time and temperature during drying (60, 70 and 80 °C). Data obtained using the simulation tool developed based on experimental data. Dots correspond to experimental values obtained at the lowest biomass density of 0.08 kg/m^2^ (thickness 0.3 cm).

This model yielded a high coefficient of determination (r^2^ = 0.9837), indicating a strong fit to the experimental data and providing a robust empirical relationship for predicting biomass quality deterioration based on drying temperature and time (Fig. 6). Using this equation, it is possible to estimate the expected deterioration as a function of drying operational conditions (Fig. 7). Previously, it was demonstrated that only when using biomass per unit surface areas below 0.08 kg/m^2^ (equivalent to a cake thickness of 0.3 cm) does the time required to completely dry the cake reduce from 9.2 h to 6.3 h and 4.3 h at temperatures of 60 °C, 70 °C, and 80 °C respectively. For these minimum drying times, the deterioration of the sample, expressed as MAE with respect to the initial sample, achieves values of 1.3, 1.4, and 1.5. This indicates that a minimum level of deterioration cannot be avoided using the proposed tray drying methodology, even under optimized conditions. Moreover, at longer drying times or higher temperatures, the deterioration is more pronounced. Therefore, it is generally not recommendable to increase the thickness of the cake by over 0.3 cm unless a relevant deterioration of biomass quality is acceptable for the intended end-use.

Summarizing, the information and models provided are valuable for optimizing microalgae tray drying, balancing efficiency and product quality. This info is crucial for precise equipment sizing, allowing them to simulate scenarios and calculate necessary drying surface areas. While tray drying is simple and adaptable for smaller scales, its large-scale industrial application faces challenges due to substantial areas and batch nature. Nevertheless, these models facilitate informed planning and investment in drying infrastructure.

## Conclusions

The study demonstrates that tray drying is a viable method for processing microalgae biomass, provided that operational parameters are carefully controlled. Through systematic variation of drying temperature (60–80 °C) and biomass layer thickness (0.3–1.0 cm), it is showed that drying performance improves significantly at higher temperatures and with thinner layers. The optimal condition—80 °C with a 0.3 cm layer—achieved complete drying in only 5 h. Kinetic modeling using a potential function accurately described the drying behavior across different temperatures, and a clear correlation was established between drying rate and water content. This enabled the development of simulation tools that can predict drying time and evaporation rate, offering a valuable tool for equipment design and process scaling. However, thermal degradation of valuable compounds limits the operational window. Despite improvements in drying kinetics, elevated temperatures and extended drying times led to significant deterioration in biomass quality, particularly of pigments and other thermolabile compounds. Spectrophotometric analysis and MAE quantification confirmed that both drying temperature and time were statistically significant factors in biomass degradation, whereas cake thickness had negligible influence on quality loss. A polynomial model was developed to predict deterioration based on drying parameters, highlighting a trade-off between process efficiency and product integrity. Even under optimal conditions, a minimum level of degradation was unavoidable, suggesting that tray drying is better suited for applications where slight quality loss is acceptable or where the targeted compounds are more thermally stable. Overall, the study provides critical insights and quantitative tools for balancing drying performance with quality preservation in microalgae biomass valorization.

## Data Availability

Data is provided within the manuscript.
